# Interaction between arbuscular mycorrhizal fungi and native soil microbiome on early stage restoration of a coal-mine soil

**DOI:** 10.1007/s00572-025-01218-3

**Published:** 2025-08-08

**Authors:** Caroline Krug Vieira, Luiz Gustavo dos Anjos Borges, Matheus Nicoletti Marascalchi, Carlos Henrique Russi, Tamiris Marandola, Karl Kemmelmeier, Cláudio Roberto Fonsêca Sousa Soares, Sidney Luiz Stürmer, Adriana Giongo

**Affiliations:** 1 Universidade Regional de Blumenau (FURB), Programa de Pós-Graduação em Engenharia Ambiental, Blumenau, Brazil; 2 Laboratory of Fungal Biology, Institute of Microbiology of the Czech Academy of Sciences, Prague, Czech Republic; 3 Pontifícia Universidade Católica do Rio Grande do Sul (PUCRS), Instituto do Petróleo e dos Recursos Naturais (IPR), Porto Alegre, Brazil; 4 Department of Mycorrhizal Symbioses, Institute of Botany, Czech Academy of Sciences, Prague, Czech Republic; 5 Instituto Nacional de Pesquisas da Amazônia (INPA), Programa de Pós- Graduação em Ecologia, Manaus, Brazil; 6 Departamento de Ciências Naturais (DCN), Universidade Regional de Blumenau (FURB), Blumenau, Brazil; 7 Universidade Federal de Santa Catarina (UFSC), Florianópolis, Brazil; 8 Leibniz Institute of Vegetable and Ornamental Crops (IGZ), Theodor- Echtermeyer-Weg 1, 14979 Großbeeren, Germany

**Keywords:** Combined inoculation, Glomeromycota, Microbiome, Simplified community, AM fungal consortia

## Abstract

The recovery of the soil ecosystem after severe disturbances, such as coal-mining activities, depends on both abiotic and biotic improvements. This study assessed the influence of arbuscular mycorrhizal (AM) fungal consortia on microbial community dynamics across two stages of soil recovery − 2 years (2Y) and 15 years (15Y) post-disturbance - using a secondary succession forest (SSR) as a reference. We analyzed bacterial community composition via 16 S rRNA gene amplicon sequencing and evaluated key soil quality indicators. While inoculation with AM fungal consortia had minimal effects on most soil parameters, significant differences were observed between recovery stages. The 15Y recovery site exhibited improved soil structure, microbial activity, and aggregate stability compared to the 2Y site, highlighting the importance of long-term restoration. However, potential overlap in ecological roles among native microorganisms likely mitigates the impact of AMF inoculation. These findings suggest that AM fungal consortia alone may not drive immediate improvements in soil quality but can contribute to microbial interactions and recovery processes over time. This study highlights the complexity of soil restoration and emphasizes the need for strategies that integrate plant cover with microbial community development to enhance long-term ecosystem stability. Further research should explore the specific roles of AM fungi and native soil microbes in promoting soil structure and accelerating recovery.

**Supplementary Information:**

The online version contains supplementary material available at 10.1007/s00572-025-01218-3.

## Introduction

Coal extraction, primarily via open-pit mining, has severe environmental consequences, including landscape alteration, the loss of native vegetation, and the formation of acid mine drainage. These disturbances contribute to soil, water, and air contamination, disrupting biological interactions and compromising essential ecological processes (Ma et al. 2023; Xu et al. 2023). Although a shift toward underground mining has been considered to mitigate such impacts, effective soil restoration remains indispensable for reversing degradation, enabling reforestation, and promoting long-term ecosystem resilience (Feng et al. 2021). Restoring soil health is a cornerstone to successful reforestation, as healthy soils regulate nutrient cycling, water retention, and microbial diversity, key factors supporting forest growth and ecosystem stability (Robinson et al. 2024).

In this context, soil inoculation with beneficial microorganisms—particularly arbuscular mycorrhizal (AM) fungi—has emerged as a promising restoration strategy. AM fungi form symbiotic associations with the roots of approximately 72% of all terrestrial plant species, exchanging carbohydrates and lipids for essential nutrients such as phosphorus and nitrogen (Smith and Read 2008; Brundrett and Tedersoo 2018). Applying Grime’s C-S-R framework (competitor‑stress tolerator‑ruderal, Grime, 1979) to AM fungi highlights that their ecological roles vary according to life‑history strategies: Acaulosporaceae family members are stress-tolerant but have lower root‑colonization rates; Glomeraceae members prioritize extensive colonization of plant roots under less stressful conditions (Hart and Reader 2002; Chagnon et al. 2013). In contrast, Gigasporaceae taxa invest in expansive extraradical mycelium and delayed sporulation, which confers a competitive advantage in resource‑rich soils (Chagnon et al. 2013). These complementary traits allow AM fungal species to enhance nutrient uptake, improving soil aggregation jointly, and influence associated microbial communities (Emmett et al. 2021; Antunes et al. 2025).

AM fungi also interact synergistically with bacteria. Their hyphae and spores create protective niches, while organic‑rich exudates function as chemoattractants and substrates for bacteria that act as cooperative partners in the AM fungi-plant symbiosis (Sangwan and Prasanna 2022; Qiu et al. 2025; Vieira et al. 2025). Due to their limited saprotrophic capabilities (Tisserant et al. 2013; Miyauchi et al. 2020; Malar et al. 2022), AM fungi interact on bacteria specialized in mineralizing organic phosphorus and nitrogen, which boosts nutrient cycling efficiency (Sbrana et al. 2022; Lu et al. 2023; Wang et al. 2024). These cooperative interactions enhance plant growth and stress resilience while aiding contaminant degradation, yielding integrated benefits for soil and plant health (Basiru and Hijri 2022; Qian et al. 2022).

Microbial consortia, including synthetic communities (SynCom) composed of selected microbial taxa under defined conditions to mimic microbiome functions, represent an emerging tool in ecological restoration. While SynCom research has primarily focused on bacteria, only a handful have explored the interactions with other soil microorganisms, such as AM fungi (Anckaert et al. 2024; Duan et al. 2024; Jin et al. 2024). A deeper understanding of AM fungal ecology and their interplay with soil bacteria is essential for enhancing the efficacy of AM fungi inoculants in restoring degraded areas (Hu et al. 2022). However, the complexity of microbial interactions across diverse ecosystems remains insufficiently understood, and further research is needed to optimize microbial consortium application in ecosystem restoration.

In this study, we used a greenhouse experiment with palisade grass (*Urochloa brizantha*) to investigate how AM fungal consortia, varying in species richness and composition, affect bacterial diversity and soil quality in soils recovering from a coal mining area and adjacent secondary succession forest. We hypothesized that (i) AM fungal consortia containing Gigasporaceae would enhance bacterial diversity and soil quality more effectively than the other combinations, and (ii) higher AM fungal consortia richness would promote beneficial bacterial taxa and improve soil quality metrics.

## Materials and methods

### Study sites, soil sampling, and characterization

**Fig. 1:**
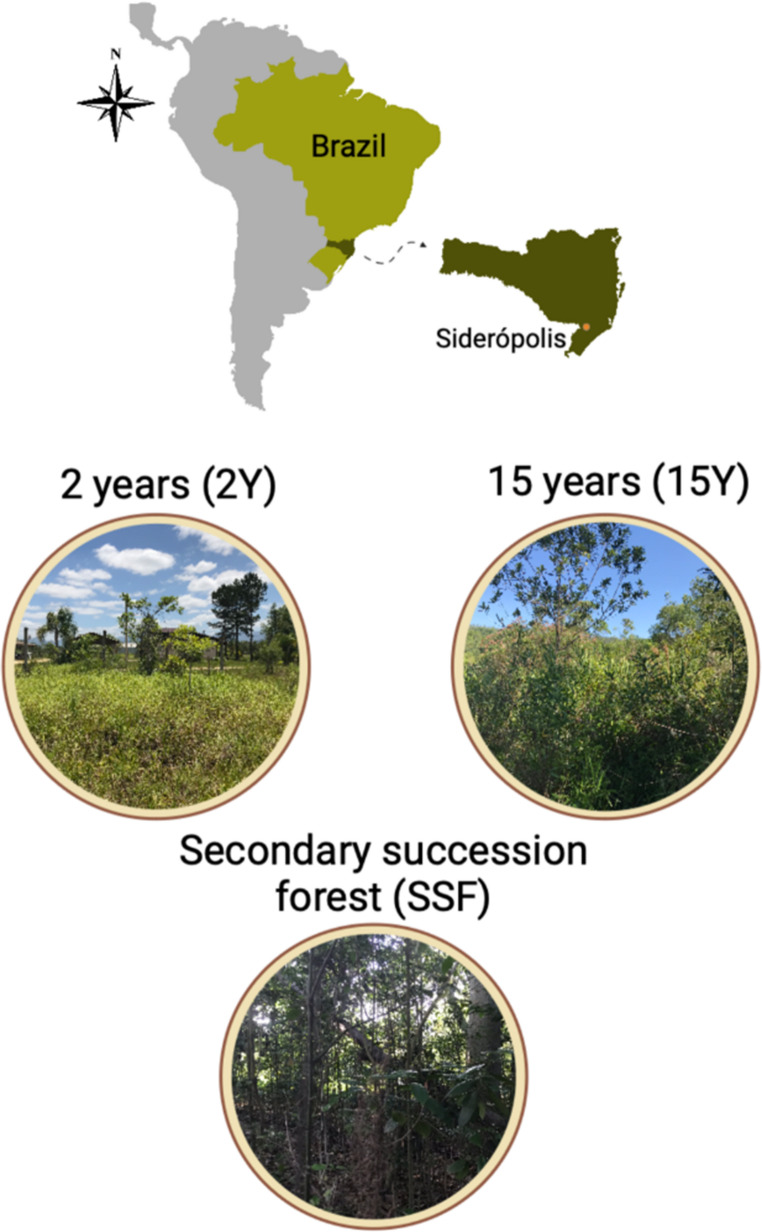
Field sampling locations in Siderópolis, Brazil, highlighting sites with distinct restoration durations: 2 years (2Y) and 15 years (15Y) post-reforestation, alongside a secondary succession forest (SSF) serving as a reference site. Scheme generated in BioRender.com

Soil samples were collected in November 2018 from two sites undergoing revegetation processes for two years (2Y) and fifteen years (15Y) in Siderópolis, Brazil [49°26’14” W, 28°35’79” S] (Fig. 1, Table S1). An adjacent secondary succession forest (SSF) in an advanced stage, not impacted by coal mining but near sites 2Y and 15Y, was chosen as a reference soil site. Local climate is classified by Köppen as Cfa (subtropical climate with a hot summer) (Alvares et al. 2013), with an annual mean temperature of 19.4 °C and 1,380 mm of precipitation. The recovery process started by removing sterile contaminating materials and topographic reconstruction of the terrain, then adding a layer of clayey soil (originating from the B horizon) from adjacent sites. Due to the low organic matter levels, 51.7 t ha^−1^ of poultry litter and 150 t ha^−1^ of peat were mixed in the soil layer. In addition, 666.7 kg ha^−1^ of P and K, 400 kg ha^−1^ of N (applied as urea) were used as chemical fertilizers, and 29 t ha^−1^ of lime was applied to raise the soil pH to 6.5. Finally, a consortium of native plants, including grasses and legumes, was used for revegetation, providing rapid soil cover, protection against erosion, and nitrogen incorporation into the soil (Vieira et al. 2022). Transplanted woody species mixed with grass at site 2Y, while woody species predominantly covered the other two sites. During the initial stages of reforestation, species from the Fabaceae family were introduced to facilitate nitrogen incorporation in 2Y and 15Y areas. Fabaceous plants were absent in the SSF site (Table S2).

Soil samples (0–10 cm depth) were randomly collected from each site using a shovel. To avoid contamination, all tools were thoroughly cleaned and sterilized with alcohol between sampling areas. Samples were then homogenized using a 4 mm sieve and stored in plastic bags. For the microcosms and the evaluation of biological characteristics, the soils were stored at 4ºC until processing. Sample aliquots were stored in liquid nitrogen for further DNA extraction. Standard physicochemical analyses were performed in the original soil after sampling, as described by Vieira et al. (2022).

### Microcosms and AM fungal consortia inoculations

The effects of AM consortia on the sporulation of AM fungi, root colonization, soil quality indicators, and bacterial diversity and composition were examined in a greenhouse experiment. Microcosms were prepared with soils from 2Y, 15Y, and SSF sites using 3 L plastic pots with drainage holes. Each treatment was performed in triplicate and involved six AM fungal consortia that varied in both species richness and taxonomic composition, comprising species from Acaulosporaceae and Gigasporaceae families and a combination of four, eight, or sixteen AM fungal species from different families (Table S3). The six AM fungal consortia comprised 22 AM fungi isolates from seven families obtained from the International Culture Collection of Glomeromycota (www.furb.br/cicg*).* The isolates are initially propagated in pure cultures maintained in a greenhouse. After confirming that the cultures are free of contaminants, the soil containing spores, roots, and hyphal fragments is stored in zip-lock bags and refrigerated at 4 °C. For microcosm inoculation, spores from each isolate were extracted using a wet sieving method (Gerdemann and Nicolson 1963), followed by centrifugation in a sucrose gradient (20% and 60%) (Jenkins 1964). Viable spores were carefully selected, counted, and then transferred into beakers containing autoclaved distilled water to produce each AM consortium, which were subsequently distributed among the respective microcosms. AM consortium 1 comprised four Acaulosporaceae species, AM consortium 2 comprised four Gigasporaceae species, and AM consortium 3 comprised two Acaulosporaceae and two Gigasporaceae species. AM consortium 4, AM consortium 5, and AM consortium 6 represent a mixture of four, eight, and sixteen AM fungal species, respectively. Species for AM consortium 4 to AM consortium 6 were randomly selected from a pool of 22 species (Table S3) following the approach used by Van der Heijden et al. (1998). Pots without inoculation were used as the control group. Each microcosm from the treatments AM consortium 1 and AM consortium 2 received a mixture of 200 spores, equally divided among four species representing each family. For treatment with AM consortium 3, two species from each family were selected for each treatment replicate, totaling 200 spores. The microcosms treated with AM consortium 4, AM consortium 5, and AM consortium 6 were inoculated with 240 spores from various species designated for each treatment. An additional 40 spores were included to ensure that each species contributed at least 15 spores to each AM fungal consortium.

The host plant used in the microcosms was palisade grass, *Urochloa brizantha* (Hochst. ex A. Rich.) R.D. Webster (syn. *Brachiaria brizantha* (A. Rich.) Stapf; Ferreira et al. 2021). Palisade grass is highly dependent on AM fungi for nutrient uptake, especially phosphorus, making it an ideal model for plant-fungal symbiosis studies. Its extensive, fibrous root system offers a large surface area for AM fungal colonization, and it thrives in nutrient-poor, acidic soils where mycorrhizal associations play a crucial role in nutrient acquisition (Álvarez-Lopeztello et al. 2019). Seeds of *U. brizantha* were disinfected with 70% alcohol for 30 s, washed with sterile distilled water, and sown in styrofoam trays in sterilized vermiculite. When seedlings were 5 cm tall, ten plants were transferred to each microcosm and irrigated with autoclaved water. A modified Hoagland’s nutrient solution containing macro- and micronutrients was applied to the microcosms every 15 days to promote plant growth.

The greenhouse experiment was conducted from December 2018 to June 2019, spanning 180 days under a completely randomized design. During the experimental period, the microcosms were maintained under natural light conditions (9.2 ± 1.9 h of daylight per day) and ambient temperatures (18 ± 3.4 ºC). At the end of the experiment, the plants were removed from the microcosms. Shoots were separated from the roots and dried at 65 °C in a forced-ventilation oven for at least 72 h to determine the shoot dry mass (SDM). Roots were cleaned under tap water, kept at 4 °C before staining, and inspected for AM fungal colonization. Part of the soil was weighed, separated in sterile plastic bags, and kept at 4 °C for soil microbiological analyses (quantities are detailed in each methodology below). For molecular analysis, the soil was stored in liquid nitrogen until processing.

### Amplicon sequencing and microbial analyses

The soil microbial communities from the original soil and greenhouse experiment were analyzed using amplicon sequencing, as previously described by Vieira et al. (2018). Total soil DNA was extracted using the DNeasy PowerSoil Kit (Qiagen), following the manufacturer’s specifications. The fragments generated by the primers 515 F and 806R (Caporaso et al. 2011) were sequenced using the Illumina MiSeq v2 (2 × 250 bp; Illumina, San Diego, USA) at Neoprospecta Microbiome Technologies (Florianópolis, Brazil). Raw data were deposited in the NCBI Sequence Read Archive (SRA) under BioProject PRJNA1105873.

Amplicon sequencing variants (ASVs) were generated using the DADA2 pipeline v.1.12.1 (Callahan et al. 2016) in R version 4.2.0 (R Core Team 2021), retaining high-quality reads longer than 100 bp with a maximum of two anticipated errors per read. A total of 903,088 quality-filtered sequences were obtained. Taxonomic assignment of ASVs was performed against the SILVA database v.138 (Quast et al. 2013) and imported into the phyloseq package (McMurdie and Holmes 2013). ASVs unassigned at the phylum level, as well as those identified as chloroplasts, mitochondria, or eukaryotes, were removed.

To normalize sequencing depth, the dataset was rarefied to the lowest number of sequences among all samples (Schloss 2024), resulting in an average of 19,900 sequences per sample. Alpha diversity was assessed using Shannon (diversity), Chao1 (richness), and Pielou (evenness) indices, which were calculated using the vegan (Oksanen et al. (2024)) and microbiome (Lahti et al. 2017) packages. Significance was assessed using Kruskal-Wallis tests followed by post hoc Wilcoxon-Mann-Whitney tests. Beta diversity was analyzed on square root transformed ASV counts using the vegan package. Bray-Curtis dissimilarity matrices were computed, and multidimensional scaling (MDS) was used to visualize variation in bacterial community composition based on 16 S rRNA gene sequences (McMurdie and Holmes 2013; Lahti et al. 2017). Differences in beta diversity centroids were evaluated using permutational multivariate analysis of variance (PERMANOVA) via the ADONIS function. Microbial composition was examined at the phylum and the lowest taxonomic levels available. Bar plots and heatmaps were generated using the phyloseq and microbiome packages. Unless otherwise stated, all graphic plots were generated using ggplot2 (Wickham 2016).

### AM fungi spore counts, identification, and AM fungal colonization

The AM fungal community from each area was characterized from triplicate samples of 50 g of soil. After the experiment was established, a 50 g subsample of soil was taken from each microcosm to extract AM fungi spores using the wet sieving method, followed by centrifugation in a sucrose gradient (20% and 60%). The total number of spores was counted and then separated by morphotypes based on size, color, and shape, and mounted on permanent slides with polyvinyl-lactic acid-glycerol (PVLG) and PVLG mixed with Melzer’s reagent (1:1, v/v). Spores were observed in a light microscope (Zeiss Axio Imager A2) and identified at genus or species level based on spore wall structure, reaction on Melzer reagent, and comparison with the original descriptions and the webpages of INVAM (https://invam.ku.edu; The University of Kansas, Lawrence, USA) and Błaszkowski (2012).

Mycorrhizal colonization was measured using 0.2 to 0.5 g of *U. brizantha* roots from each microcosm, which were removed from the host plants and stained following the method described by Grace and Stribley (1991). Roots were washed, dried, and immersed in 10% KOH solution at room temperature for 22 h. Afterward, the roots were heated for 1 min in a microwave and washed with tap water to remove the KOH completely. Roots were then immersed in acetic acid for 5 min. The acid was removed, and the roots were covered with a 0.05% Trypan blue solution and heated for 45 s in a microwave. Lastly, the roots were washed and immersed in glycerol, water, and HCl solution for 5 min. From each sample, 50 root fragments were randomly selected and mounted on microscope slides with PVLG. AM fungal colonization was measured as described by McGonigle et al. (1990) under a light microscope (Zeiss Axio Imager A2).

Alpha diversity of AM fungi communities based on spore identification was assessed using Hill numbers, while beta diversity differences were explored using SIMPER analysis to identify which taxa contributed most to the dissimilarity between samples. These analyses were performed using the *vegan* (Oksanen et al. 2024) and *microbiome* (Lahti et al. 2017) packages.

### Soil quality indicators evaluation

For β-glucosidase, 1 g of soil samples was placed in a 50 mL plastic tube, and 0.25 mL of toluene, 4 mL of modified universal buffer (MUB; pH 6.0), and 1.0 mL of PNG solution were added. The tubes were shaken and incubated for 1 h at 37 °C. After incubation, 1.0 mL of CaCl_2_ and 4 mL of tris hydroxymethyl aminomethane solution at pH 12 were added to the samples. For acid phosphatase, 1-g soil samples were placed in a 50 mL plastic tube, and 0.25 mL of toluene, 4 mL of modified universal buffer (MUB at pH 6.5), and 1.0 mL of PNP solution were added. Tubes were shaken and incubated for 1 h at 37 °C. After incubation, 1 mL of CaCl_2_ and 4 mL of NaOH were added to the samples. The supernatant solution was filtered, and the intensity of the color was measured at 410 nm (Spectrophotometer SP 2100 UV-Vis, Shanghai Spectrum). The results were expressed in µg of p-nitrophenol released by g^−1^ soil h^−1^, and the concentration of PNG and PNP was determined based on standard calibration curves. Assessment of soil aggregate stability, easily extractable (EE-GRSP) and total glomalin-related soil protein (T-GRSP), determination of fluorescein diacetate hydrolysis (FDA), and microbial respiration, soil microbial biomass carbon (C-MB), and metabolic quotient (*q*CO_2_) were processed as described by Vieira et al. (2022).

### Statistical analyses

Statistical analyses were conducted to assess the impact of different treatments on diversity indices in soils from 2Y, 15Y, and SSF sites. The Kruskal-Wallis test was used for non-normally distributed data, while ANOVA was applied for normally distributed data. Pairwise differences between treatment groups and specific taxa abundances were evaluated using the Wilcoxon pairwise test (for non-parametric data) or the t-test (for parametric data). Beta diversity was assessed using PERMANOVA (ADONIS) with 10,000 permutations to evaluate dissimilarities among samples. Two-way ANOVA and principal component analysis (PCA) were employed to examine the effects of sample location, treatment, and their interaction on spore counts, colonization, and soil quality indicators. Contrast analysis using the *lm* function was performed to compare each treatment individually with the control at each location. Before analysis, total spore counts were log-transformed [log10(x + 1)], and AM fungal colonization data were transformed using the arcsine square root transformation of the proportion of colonization. The impact of treatment on the bacterial community was assessed by identifying clusters of correlation using Spearman correlation analysis, with significance set at *p* < 0.01. Normality and homogeneity of variance were assessed using the Shapiro-Wilk and Levene’s tests, respectively. Parameters violating these assumptions were log-transformed. PCAs were conducted using the *vegan* package, with significance determined through 10,000 permutations. All analyses were performed in R (R Core Team 2021), with statistical significance set at *p* < 0.05.

## Results

### Soil attributes and parameters

**Fig. 2:**
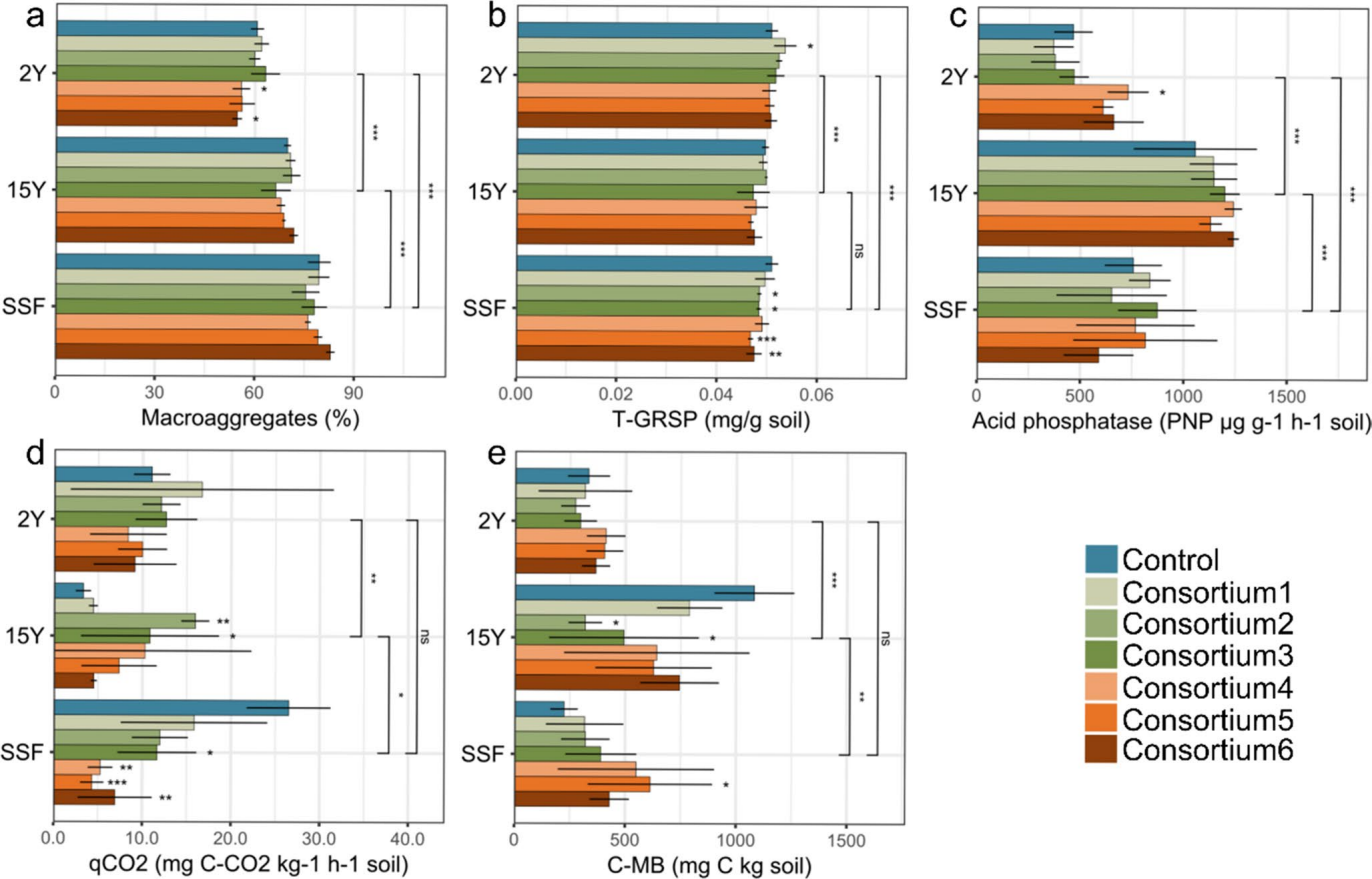
Soil parameters post-greenhouse experiment. Soil attributes measured include (**A**) percentage of soil macroaggregates, (**B**) total glomalin-related soil protein (T-GRSP), (**C**) acid phosphatase activity measured as *p*-nitrophenyl phosphate (PNP), (**D**) metabolic quotient (*q*CO_2_), and (**E**) microbial biomass carbon (C-MB). Comparisons were made between control samples and various treatments: AM consortium1 (Acaulosporaceae), AM consortium2 (Gigasporaceae), AM consortium3 (Acaulosporaceae and Gigasporaceae), AM consortium4 (AM fungal richness, *n* = 4), AM consortium5 (AM fungal richness, *n* = 8), and AM consortium6 (AM fungal richness, *n* = 16). Analyses were conducted within each site: 2 years post-reforestation (2Y), 15 years post-reforestation (15Y), and a secondary succession forest (SSF) was used as a reference. Statistical significance between treatments and sites was assessed using ANOVA, with significance levels denoted by asterisks (* *p* < 0.05; ** *p* < 0.01; *** *p* < 0.001). Vertical bars represent contrasts between sites (Two-way ANOVA- Contrast analysis), and standard errors are depicted as black lines

AM consortium 4 (68%) and consortium 6 (66%) showed reduced macroaggregate percentages in 2Y compared to the control (75%) (*p* = 0.047 and *p* = 0.016, respectively) (Fig. 2A). For 15Y and SSF, macroaggregate percentages did not significantly differ among treatments (*p* > 0.05).

T-GRSP content ranged from 0.046 to 0.053 mg g^−1^ soil, with 2Y showing the highest amount and differing significantly from 15Y and SSF (*p* < 0.001; Fig. 2B). In 2Y, AM consortium 1 showed the highest T-GRSP levels (0.0535 mg g^−1^ soil, *p* = 0.034), while in SSF, AM consortium 2, AM consortium 3, AM consortium 5, and AM consortium 6 exhibited reduced T-GRSP levels compared to the control (*p* < 0.05, Table S4). EE-GRSP content and microbial basal respiration did not significantly vary between areas or treatments (*p* > 0.05; Fig. S1D, Table S4).

Acid phosphatase activity was significantly higher in 15Y (1054.4 µg p-nitrophenol g^−1^ h^−1^ soil) compared to 2Y (462.7 µg p-nitrophenol g^−1^ h^−1^ soil) and SSF (756.8 µg p-nitrophenol g^−1^ h^−1^ soil) (*p* < 0.001; Fig. 2C; Table S4). Among the treatments, AM consortium 4 significantly increased acid phosphatase activity in 2Y compared to the control (*p* = 0.015). FDA activity was highest in 2Y (45.5 µg g^−1^ h^−1^ fluorescein soil), significantly differing from 15Y and SSF (*p* < 0.01), but showed no significant differences between treatments. β-glucosidase activity varied from 1040.4 to 391.4 µg p-nitrophenol g^−1^ h^−1^ soil across sites, with no significant differences observed (*p* > 0.05) except by AM consortium 6 treatment in 2Y, which displayed a significantly lower β-glucosidase activity than the control (*p* = 0.003).

*q*CO2 was significantly lower in 15Y (3.3 mg C-CO_2_ kg h^−1^ soil) compared to 2Y and SSF (11.0 and 26.5 mg C-CO_2_ kg h^−1^ soil, respectively; *p* < 0.05; Fig. 2D). In 15Y, AM consortium 2 and AM consortium 3 showed significantly higher *q*CO_2_ values compared to the control (*p* < 0.05), while in SSF, AM consortium 3, AM consortium 4, AM consortium 5, and AM consortium 6 exhibited significantly lower *q*CO2 compared to the control (*p* < 0.05).

In contrast, C-MB was significantly higher in 15Y (1081.3 mg C kg soil) than in 2Y and SSF (332.9 and 224.2 mg C kg soil, respectively; *p* < 0.01; Fig. 2E). In 15Y, AM consortium 2 and AM consortium 3 showed significantly lower C-MB levels than the control (*p* < 0.05), while in SSF, AM consortium 5 exhibited significantly higher C-MB compared to the control (*p* = 0.018).

### Plant and mycorrhiza parameters

The plant shoot dry mass (SDM) ranged from 6.7 g (AM consortium2) to 10.9 g (Control) in the 2Y soil and from 7.0 g (AM consortium5) to 8.9 g (AM consortium6) in the 15Y soil, but no overall differences were found between the two areas (*p* = 0.47) (Table S4). However, the highest SDM values were recorded with the SSF soil, ranging from 8.2 g (Control) to 15.2 g (AM consortium5), which showed a significant increase compared to both 2Y (*p* = 0.02) and 15Y (*p* = 0.001). The treatment with AM consortium5 in SSF also showed the highest SDM values compared to the control (*p* = 0.02) and SynCon5 from 15Y (*p* = 0.01).

**Fig. 3:**
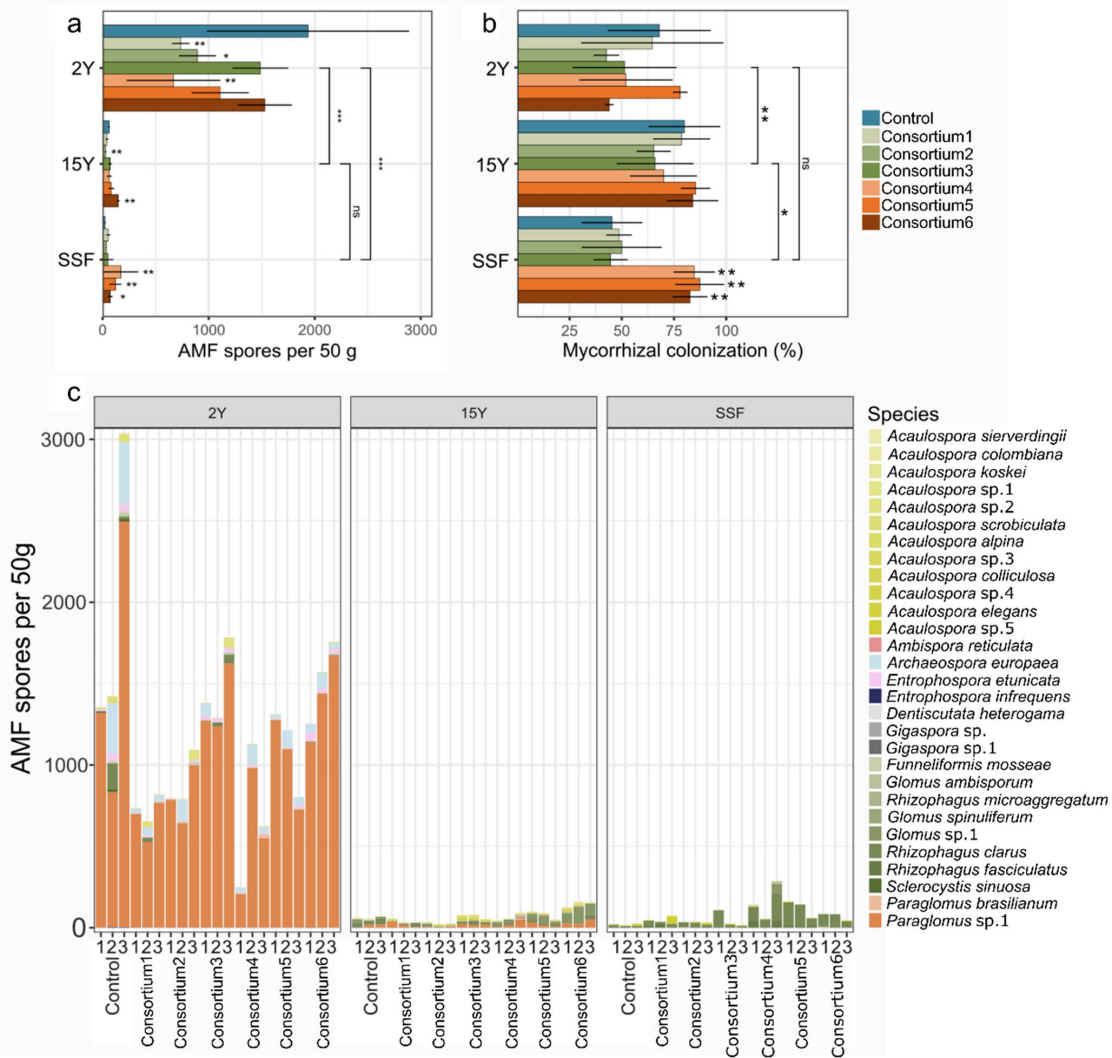
Total AM spore count and AM fungal colonization of *Urochloa brizantha* across mining sites with short-term recovery (2Y), long-term recovery (15Y), and a secondary succession forest (SSF) observed post-greenhouse experiment. (**A**) Total number of AM spores per 50 g of soil (mean ± standard deviation); (**B**) Percentage of AM fungal colonization in *U. brizantha* roots (mean ± standard deviation); (**C**) Total AM spore count and species recovered from each microcosm. Comparisons between control and treatment samples [AM consortium1 (family Acaulosporaceae); AM consortium2 (family Gigasporaceae); AM consortium3 (families Acaulosporaceae and Gigasporaceae); AM consortium4 (AM richness, *n* = 4); AM consortium5 (AM richness, *n* = 8); AM consortium6 (AM richness, *n* = 16)] at each site (2Y, 15Y, and SSF) were performed using Two-way ANOVA with contrast analysis. Vertical bars represent species’ presence across sites. Statistical significance is denoted by asterisks (* *p* < 0.05; ** *p* < 0.01; *** *p* < 0.001). Standard errors are shown as black lines. Data is presented in triplicate

The total number of AM fungal spores across treatments varied from 62 to 1936 per 50 g of soil (Table S4, Fig. 3A). Spore counts were significantly higher in the 2Y site (1936 spores/50 g soil) compared to the 15Y and SSF sites (61 and 21 spores/50 g soil, respectively) (Two-way ANOVA; *p* < 0.001). In the 2Y site, inoculation with AM consortium1, AM consortium2, and AM consortium4 significantly reduced spore numbers compared to the control (*p* = 0.015, *p* = 0.047, *p* = 0.002, respectively). In 15Y, AM consortium1 and AM consortium2 also decreased spore counts (*p* = 0.049, *p* < 0.001), while AM consortium6 increased them (*p* = 0.003). In SSF, inoculation with AM consortium4, AM consortium5, and AM consortium6 led to a significant increase in spore numbers compared to the control (*p* = 0.006, *p* = 0.004, *p* = 0.021, respectively).

Mycorrhizal colonization in *U. brizantha* roots ranged from 42.7 to 87.3%. Colonization was higher in 15Y (80%) compared to 2Y (68%) and SSF (45.3%) (*p* = 0.002 and *p* = 0.045, respectively), but no significant difference was observed between 2Y and SSF (*p* = 0.438) (Fig. 3B). In SSF, inoculation with AM consortium4, AM consortium5, and AM consortium6 significantly increased mycorrhizal colonization compared to the control (*p* = 0.003, *p* = 0.001, *p* = 0.003, respectively).

A total of 29 AM fungi morphotypes were identified after the experiment, with 10 species assigned to 8 families within the Glomeromycota (Fig. 3C). Species occurrence varied by site and inoculation treatment, with several exclusive species, 6, 4, and 3, detected in 2Y, 15Y, and SSF, respectively. Total species richness ranged from 13 in SSF to 20 in 2Y. The most common species were *P. brasilianum*, *Paraglomus* sp.1, and *A. europaea* in 2Y; *Glomus* sp., *Paraglomus* sp.1, and *R. clarus* in 15Y; and *R. clarus*, *R. fasciculatus*, and *Glomus* sp.1 in SSF.

Several AM fungal species from the initial soil samples and AM fungal consortia, including *A. colombiana*, *A. scrobiculata*, *D. heterogama*, *R. clarus*, *E. etunicata*, and *P. brasilianum*, persisted until the end of the experiment across the different sites. However, species from AM consortium2 and AM consortium3 did not remain in the soil at the 2Y site, AM consortium2 did not persist in 15Y, and AM consortium1 and AM consortium3 did not persist in SSF (Table S2; Table S5).

For the 2Y and SFF areas, Hill index values among consortia were lower than the values found for the control (Table S6). Conversely, the Hill value for the control was similar to those of consortia 1 and 4 in the 15Y area (Table S6), while the values for the other consortia were either higher (consortia 2 and 3) or lower (consortia 5 and 6). The SIMPER analysis calculated only for the 15Y revealed that *R. clarus* contributed the most for the difference in the Hill index in consortium1, consortium2, and consortium4 (18–27.5%), followed by *Paraglomus* sp. 1 in consortium1 and consortium2 (12–13.3%), and *Glomus* sp. 1 in consortium4 (12.1%). For consortium3, *Acaulospora* sp. 2 was the species that contributed the most for the difference (11.50%) followed by *R. clarus* (11.10%) (Table S7).

### Correlation of AM fungal inoculation and soil quality indicators

Principal component analysis (PCA) assessed how different AM fungal inoculation treatments correlated with soil quality indicators. The first two RDA axes explained 55.5%, 55.7%, and 59.8% of the variability in the data for the 2Y, 15Y, and SSF sites, respectively, suggesting that AM treatments have a stronger effect on soil quality in short-term recovery sites.

**Fig. 4:**
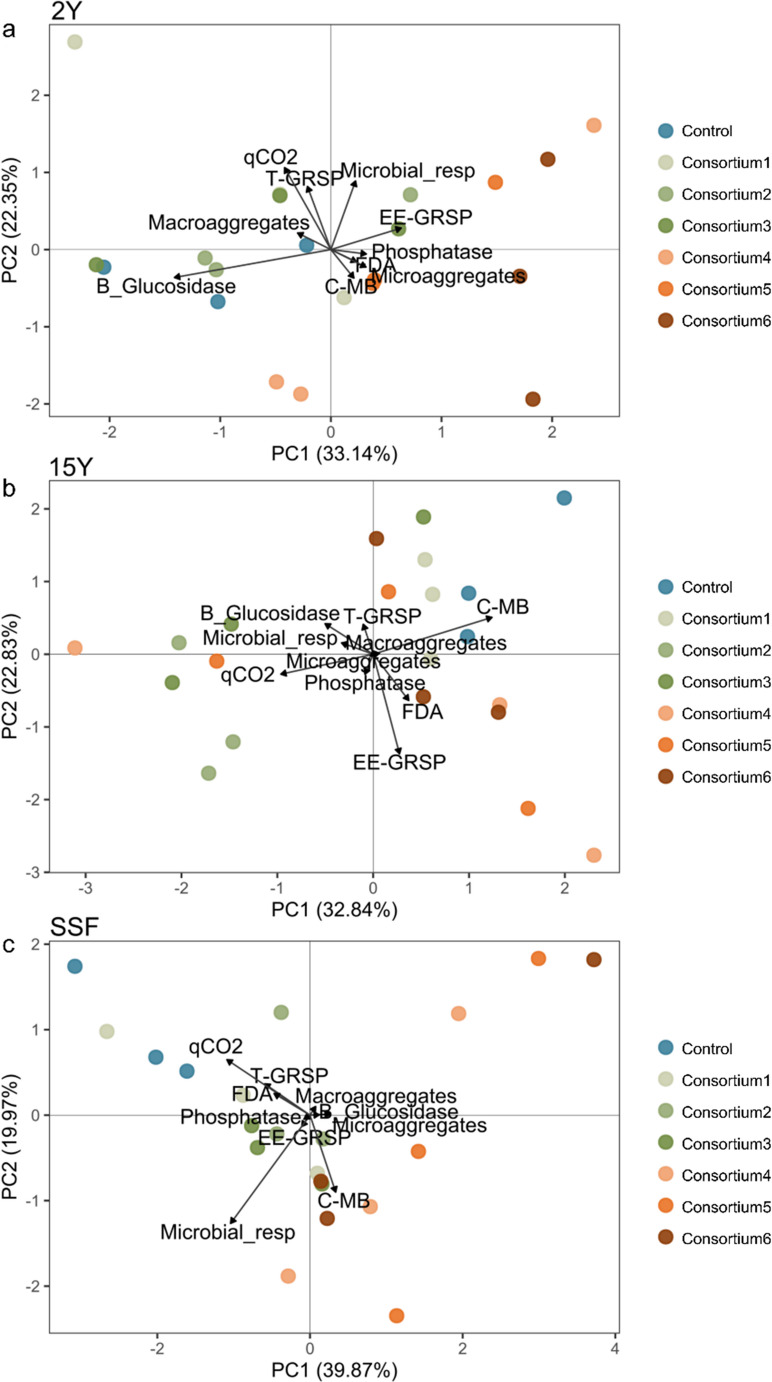
Principal Component Analysis (PCA) and Multivariate Analysis of Variance (MANOVA) were used to analyze how AM inoculation treatments relate to soil quality indicators in soils from mining sites with short-term recovery (2Y), long-term recovery (15Y), and secondary succession forest (SSF). Comparisons are made between control and treatment samples, which include AM consortium1, family Acaulosporaceae; AM consortium2, family Gigasporaceae; AM consortium3, families Acaulosporaceae and Gigasporaceae; AM consortium4, AM richness (*n* = 4); AM consortium5, AM richness (*n* = 8); AM consortium6, AM richness (*n* = 16), within each site, (**A**) short-term recovery (2Y); (**B**) long-term recovery (15Y), (**C**) secondary succession forest (SSF)

At the 2Y site, AM consortium2 and AM consortium3 were positively linked with microbial basal respiration and glomalin-related soil proteins (MANOVA; *p* < 0.001) (Fig. 4A). In contrast, at the 15Y site, AM consortium1, AM consortium5, AM consortium6, and the control were associated with macroaggregates, total glomalin-related soil proteins, and soil microbial biomass carbon (MANOVA; *p* < 0.001) (Fig. 4B). For the SSF site, AM consortium4, AM consortium5, and AM consortium6 formed a distinct group from the controls, although the differences among treatments were not significant (MANOVA; *p* = 0.084) (Fig. 4C).

### The effect of AM fungal inoculation on soil bacteria

**Fig. 5:**
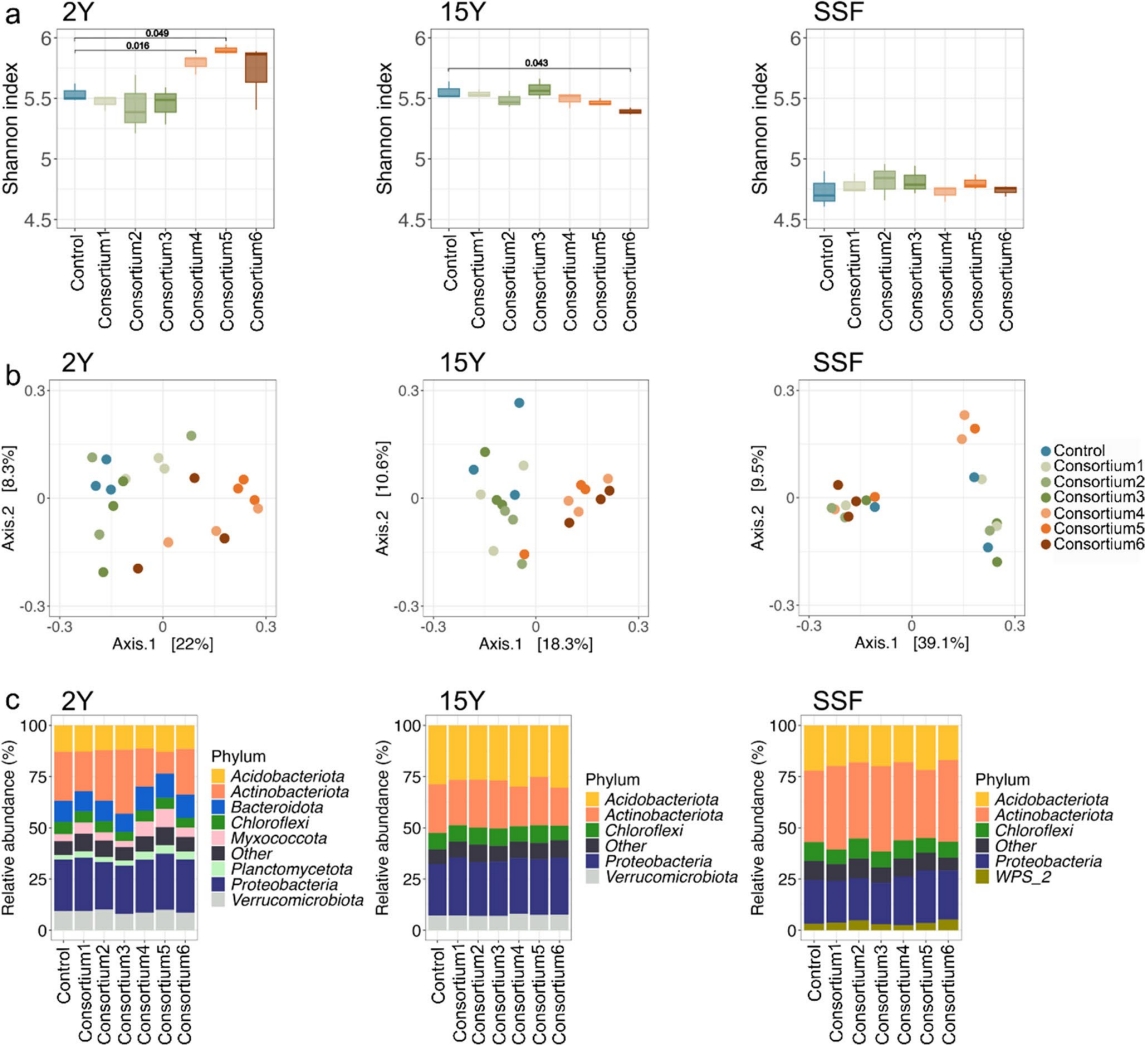
Analysis of microbial diversity and composition based on 16 S rRNA gene amplicon sequencing in different treatments, AM consortium1 (Acaulosporaceae), AM consortium2 (Gigasporaceae), AM consortium3 (Acaulosporaceae and Gigasporaceae), AM consortium4 (AM fungal richness, *n* = 4), AM consortium5 (AM fungal richness, *n* = 8), and AM consortium6 (AM fungal richness, *n* = 16), within each site, short-term recovery (2Y), long-term recovery (15Y), and a secondary succession forest (SSF). (**A**) Alpha diversity is represented by Shannon indices, comparing diversity across different treatments to control samples. Statistical significance was assessed using the Mann-Whitney U test with *p*-values adjusted by the Bonferroni method. Standard errors are shown as vertical black lines. (**B**) Beta diversity displays differences in microbial community composition among treatments using multidimensional scaling (MDS). (**C**) Relative abundance (%) of the most prevalent phyla in the soil is shown. Taxa with fewer than 350 sequences in less than three samples are grouped as “Other”

The inoculation with AM consortium4 and AM consortium5 significantly increased microbial community diversity compared to control samples (Control vs. AM consortium4, *p* = 0.016; Control vs. AM consortium5, *p* = 0.005). No significant differences were found between the control and other inoculation treatments (*p* > 0.05; Fig. 5A). Bacterial beta diversity, assessed by Bray-Curtis dissimilarity, was significantly influenced by AM fungal inoculation in 2Y and 15Y soils but not in SSF (PERMANOVA; 2Y, R^2^ = 0.18, *p* = 0.015; 15Y, R^2^ = 0.19, *p* = 0.001; SSF, R^2^ = 0.14, *p* > 0.05) (Fig. 5B).

The bacterial phyla Acidobacteria, Actinobacteria, Chloroflexi, and Proteobacteria were detected in all soils. In 2Y soils, Bacteroidota, Myxococcota, and Verrucomicrobia also met the thresholds, while in 15Y soils, only Verrucomicrobiota surpassed the threshold (Fig. 5C). These phyla were observed in all treatments.

In 2Y soils, AM consortium5 treatment increased taxa from Vicinibacteraceae (Acidobacteriota) and Pedosphaeraceae (Verrucomicrobiota) compared to controls (*p* = 0.027 and *p* = 0.021, respectively). Conversely, it decreased taxa from *Allokutzneria*, *Kribella*, *Lechevalieria*, and *Streptomyces* (Actinobacteriota) (*p* = 0.026, *p* < 0.001, *p* = 0.002, and *p* < 0.001, respectively). *Streptomyces* were also decreased when 2Y soils were treated with AM consortium 1 (*p* = 0.046), along with a taxon from Gaiellales (*p* = 0.032). *Candidatus* Udaeobacter (Verrucomicrobiota) is decreased in treatments with AM consortium4, AM consortium5, and AM consortium6 (*p* = 0.049, *p* = 0.044, and *p* = 0.046, respectively).

In 15Y soils, the AM consortium6 treatment increased a taxon from the Subgroup 2 (Acidobacteriota) (*p* = 0.047). AM consortium5 increased *Acidibacter* (Proteobacteria) (*p* = 0.038), and AM consortium1 and AM consortium4 increased Pedosphaeraceae (Verrucomicrobiota) (*p* = 0.040 and *p* = 0.024, respectively). AM consortium4 decreased *Conexibacter* (Actinobacteriota) (*p* = 0.005), while AM consortium5 and AM consortium6 decreased Xanthobacteraceae (Proteobacteria) (*p* = 0.016 and *p* = 0.015, respectively). No significant differences were observed in SSF soils with the treatments (*p* > 0.05).

**Fig. 6:**
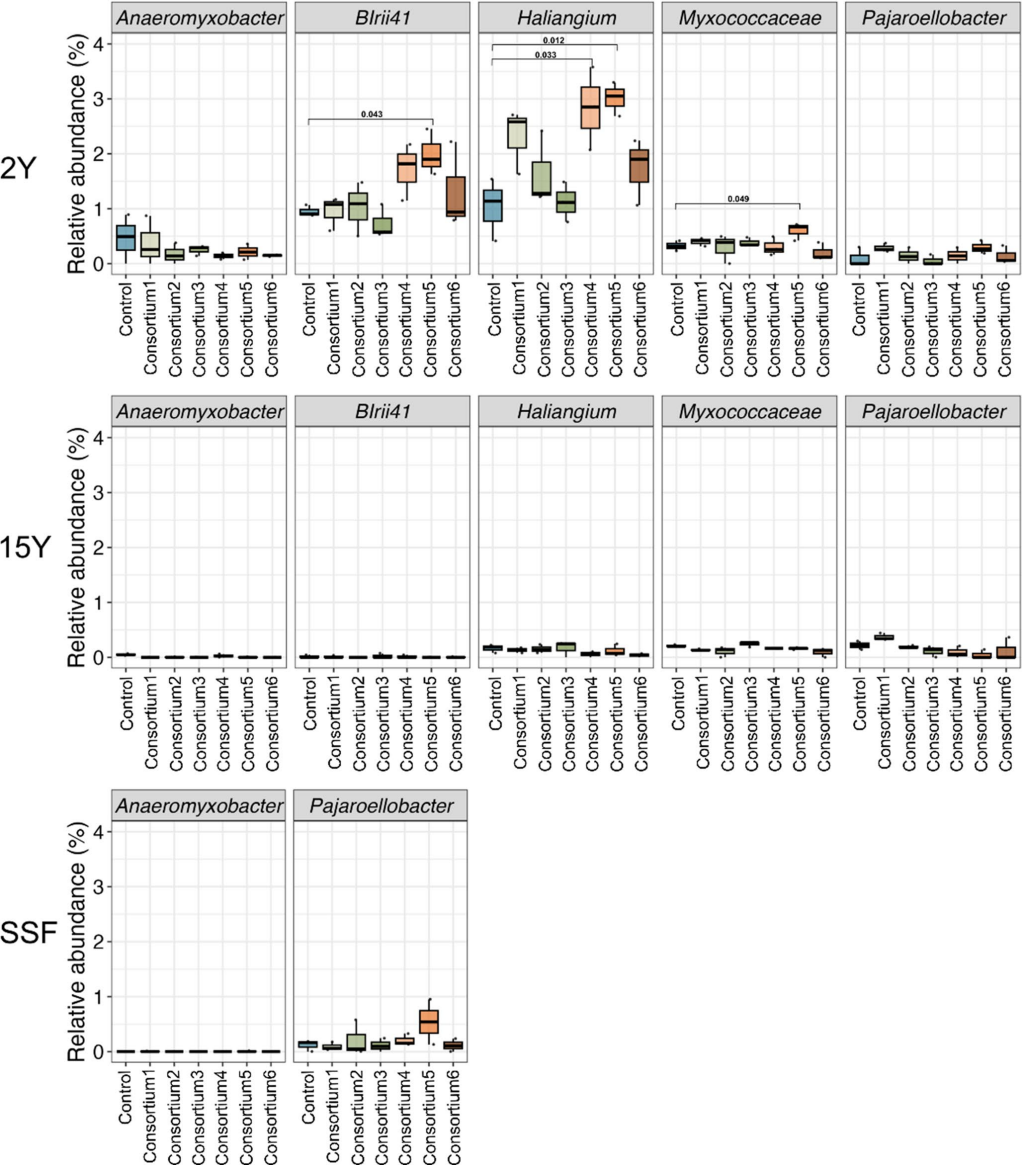
Relative abundance of Myxococcota across different sites (2Y, 15Y, and SSF) and treatments, AM consortium1 (Acaulosporaceae), AM consortium2 (Gigasporaceae), AM consortium3 (Acaulosporaceae and Gigasporaceae), AM consortium4 (AM fungal richness, *n* = 4), AM consortium5 (AM fungal richness, *n* = 8), and AM consortium6 (AM fungal richness, *n* = 16)

The phylum Myxococcota was more abundant in control and treatment samples from 2Y soils (Figure S1). In 2Y soils, Myxococcota is increased significantly in AM consortium1, AM consortium4, and AM consortium5 compared to controls (*p* = 0.029, *p* = 0.004, and *p* = 0.003, respectively) (Fig. 6A). In contrast, in 15Y soils, Myxococcota is decreased in treatments with AM consortium4, AM consortium5, and AM consortium6 in 15Y soils (*p* = 0.028, *p* = 0.048, and *p* = 0.013, respectively). No significant changes were observed in SSF soils (*p* > 0.05).

In 2Y soils, 24 Myxococcota taxa were identified, compared to 10 taxa in 15Y and three in SSF. *Pajaroellobacter* and *Anaeromyxobacter* were found in all soil types, but no taxa were shared between 2Y and SSF (Fig. 6B). The most abundant taxa included *Anaeromyxobacter*, *Haliangium*, *Pajaroellobacter*, and two other taxa from the orders Polyangiales (BIrii41) and Myxococcaceae (Fig. 6C). In 2Y soils, AM consortium5 significantly increased the abundance of Myxococcota taxa, particularly for BIrii41, *Haliangium*, and Myxococcaceae (*p* = 0.043, *p* = 0.012, and *p* = 0.049, respectively). *Haliangium* also increased in AM consortium4 (*p* = 0.033). No significant differences were found in 15Y and SSF soils (*p* > 0.05).

## Discussion

Soil microorganisms play a crucial role in ecosystem restoration, serving as indicators of recovery progress and offering insights for future restoration strategies. While evidence shows microbial groups involved in symbiotic relationships with AM fungi (Zhou et al. 2023; Wang et al. 2024), the effects of different AM fungal community compositions on microbial populations are not fully understood. This study evaluated the influence of AM fungal consortia with varying species richness and composition on soil quality parameters and bacterial communities in post-coal mining and a reference area.

### Limited influence of AM fungal consortia on soil attributes and parameters

Soil quality improvements in this study were primarily associated with the distinct recovery stages of the sites (2Y vs. 15Y) rather than with fungal treatments. Indicating that natural processes and the implementation of revegetation techniques, rather than introducing particular AM fungal families (e.g., Acaulosporaceae or Gigasporaceae consortia), were more important for enhancing soil attributes. Although some changes in the microbial taxonomic composition were observed, the resilience of the microbial communities likely allowed them to maintain key microbial processes (Nunes et al. 2018; Chen et al. 2022).

The highest richness of AM fungal species in the microcosms was observed in 2Y and 15Y. The soil was amended with lime and organic matter to restore these sites, woody seedlings were transplanted, and a mixture of grass species was sown. Consequently, AM fungal propagules may have been introduced during plant transplantation, and, together with liming, organic matter addition, and fertilization, these treatments likely enhanced overall soil-microbiota activity (De Souza et al. 2013; Lu et al. 2023). Contrary to our hypothesis, native fungi species dominated over the inoculated ones, possibly due to competition and suppression by native communities present in each area (Basiru and Hijri 2022). This pattern is consistent with the diversity-invasibility hypothesis (DIH), which predicts that more diverse communities resist the establishment of newcomers (Elton 1958; Van Elsas et al. 2012). The greater native AM fungal diversity in 2Y may have impeded the establishment of the inoculated consortia.

In the initial soil samples, *E. etunicata* and *P. brasilianum* were detected at 2Y, *A. scrobiculata* and *R. clarus* at 15Y, and *R. clarus* at SSF, indicating the persistence of these native taxa throughout the experiment. Additionally, most of the inoculated species that remained in the soil until the end of the study belonged to the Acaulosporaceae and Paraglomeraceae families. These stress-tolerant fungi and ruderal, commonly associated with disturbed environments and early-stage recovering areas, exhibited high spore production (especially *Paraglomus* sp. in 2Y) and produced fewer extra and internal root structures, reinforcing their resilience and competitive advantage (Chagnon et al. 2013; Lopes et al. 2016; Malicka et al. 2022). They regenerate rapidly through spores, enhancing their persistence (Weber et al. 2019; Cahyaningtyas and Ezawa 2024).

The dominance of *Paraglomus* sp. was crucial for the high spore density observed in 2Y, since members of the family Paraglomeraceae allocate most of their resources to spore production rather than root structures (Säle et al. 2021). In microcosms inoculated exclusively with species from the Acaulosporaceae family (AM fungal consortium1) or the Gigasporaceae family (AM fungal consortium2), total spore numbers were significantly lower than in the 2Y control. These families, known for their competitive and stress-tolerant life strategies, may suppress spore production of other species through competition for space, carbon, or colonization sites (Lekberg et al. 2007). Although few spores from these families were detected, they may persist primarily as extraradical hyphae or internal structures not captured by spore counts. In SSF, low native species richness and the reduced prevalence of pioneer taxa such as *Paraglomus* sp. may have reduced competitive pressure, potentially enabling the successful establishment of the inoculated AM fungi in the microcosms (Verbruggen et al. 2013). Treatments involving AM fungal consortia with higher species richness led to greater AM fungal colonization, reinforcing that more diverse fungal communities are better suited to colonize and persist in stable environments. The “priority effect,” where the order of AM fungal colonization shapes the structure of root-associated fungal communities, can effectively prevent or reduce colonization by later fungi (Werner and Kiers 2015). In addition, AM fungal colonization in plants can be facilitated by the hyphospheric bacteria, such as *Streptomyces* (abundant in SSF). This specific bacterial genus promotes elongation and branching of AM fungi hyphae (Duan et al. 2024).

Inoculation with an AM fungal consortium composed exclusively of Gigasporaceae species did not significantly improve soil quality compared with other families. Gigasporaceae, known for its high phosphorus absorption and extensive mycelium, was expected to enhance soil physical properties and stimulate specific microorganisms (Hart and Reader 2002; Chagnon et al. 2013; Zhang et al. 2024). Factors such as spore dormancy, environmental requirements for germination, and competition from native fungi likely limited their spore production. The persistence of fungal species in the microcosms has caused specific changes in soil quality indicators. In 2Y, the increase in acid phosphatase concentration observed in the AM consortium4 treatment may have been stimulated by the microbiome in the hyphosphere of *Paraglomus* sp.1 and *P. brasilianum*. As aforementioned, 2Y showed a high abundance of *Haliangium*, which was even more predominant in AM consortium4. Wang et al. (2023) had already observed a positive association between this bacterium and the fungus *Paraglomus* sp., both abundant in 2Y. The increase in acid phosphatase activity observed in 2Y may have resulted from the predatory behavior of *Haliangium*, promoting the decomposition of organic matter and increasing phosphorus availability in the soil (Zhang et al. 2023).

Generally, the persistence of AM fungi in SSF was predominantly associated with the family Glomeraceae. In AM consortium5, the species *R. clarus* stood out as the most abundant and persisted until the end of the experiment. This fungal combination has stimulated the development of the microbial community, both through its abundance and the increase in microbial biomass carbon, by facilitating nutrient absorption and creating a more favorable environment for microorganism development. The rapid growth of Glomeraceae species may accelerate nutrient absorption, increasing the deposition of photosynthetic carbon, the accumulation of soil organic carbon, and the regular production of fungal necromass (Horsch et al. 2023). This factor may have contributed to reducing or stabilizing the metabolic quotient, indicating more efficient carbon utilization and increased nutrient availability, reducing the necessity for excessive microbial respiration (Clayton et al. 2021). Microbial resilience and diversity of AM fungal communities, particularly those belonging to the ruderal lifestyle, such as the Glomeraceae and Paraglomeraceae families, may play a vital role in enhancing microbial activity and improving soil quality. These patterns were further supported by the Hill diversity index and SIMPER analysis, which emphasized the prominence of these taxa and their contributions to community structure.

### Do AM fungal consortia influence biotic microbial communities?

The inoculation of AM fungal consortia notably influenced bacterial microbiomes, especially in the younger recovery soils (2Y and 15Y). Consortia with higher AM fungal diversity significantly enhanced bacterial richness compared to controls, and contrary to our first hypothesis, these effects appear to be more strongly linked to the overall species richness of the consortia than to the presence of specific families such as Gigasporaceae or Acaulosporaceae. Although our spore-based inoculation method does not allow precise identification of the particular AM fungal taxa responsible for influencing bacterial communities, the inclusion of multiple AM fungal species may have broadened the spectrum of microbial interactions and contributed to the selective structuring of the hyphosphere bacterial community (Zhou et al. 2020; Luthfiana et al. 2021; Lahrach et al. 2024). This microbial richness modulation effect was most pronounced in 2Y soils, where bacterial composition was significantly impacted. These results support that AM fungi can indirectly alter bacterial communities by enhancing nutrient availability or creating favorable niches (He et al. 2024; Zhang et al. 2024). In contrast, no significant shifts were detected in SSF soils, suggesting that, as mature microbial ecosystems, the influence of external inoculants diminishes. Compared to stressed environments, the microbiota may have undergone less alteration and recruitment, resulting in a reduced need to support the ecosystem or enhance the microenvironment for plant growth (Nuccio et al. 2013; Qin et al. 2022; Wei et al. 2023).

The response of Myxococcota to AM consortia inoculation was particularly striking. In 2Y soils, AM consortium5 significantly increased several Myxococcota taxa, including BIrii41, *Haliangium*, and Myxococcaceae, which are known for their predatory behavior and role in organic matter turnover (Petters et al. 2021). It might suggest that AM fungi support microbial decomposer functions in early recovery, facilitating organic matter breakdown and nutrient cycling (Duan et al. 2024; Faghihinia et al. 2024). AM fungal inoculation has a stronger effect in early succession stages, such as in 2Y and 15Y soils, where microbial communities are more dynamic and responsive to external influences. In contrast, SSF soils showed minimal changes as the ecosystems may be closer to stability.

AM fungal consortia can influence microbial communities, particularly in early recovery. The interaction between AM fungi and bacteria is most pronounced in the early stages of soil recovery when microbial communities are still developing toward equilibrium. In environments under advanced succession, like SSF, microbial communities may have already achieved stability and been less susceptible to external inoculation strategies.

These results underscore the complexity of soil microbiome dynamics in response to AM fungal inoculation. While the effects were often subtle or context-dependent, the data support that AM fungi, exceptionally diverse consortia, can shape microbial communities in early recovery soils. We acknowledge the limited temporal scope and microcosm-based nature of this study; however, it provides valuable insights into the interplay between fungal inoculants and native microbial communities. This aspect will be further explored in future research, expanding on these findings through larger-scale and long-term restoration trials.

## Conclusion

AM fungal consortia had a limited direct impact on soil quality indicators; however, the native microbiome played a central role in driving recovery. The 15Y site showed significant improvements in soil structure and microbial activity, approaching the conditions of the SSF site. Nevertheless, microbial communities have not yet fully recovered, emphasizing the need for long-term strategies that support plant cover and microbial regeneration. Future research should explore the lasting impacts of AM fungal inoculation in combination with other restoration strategies, while also addressing challenges such as competition with native fungi. A deeper understanding of the microbial contributions to soil structure and ecosystem processes will be crucial for restoring disturbed environments. Although baseline data on microbial communities were unavailable at the beginning of the study, the experimental design accounted for site-specific variation through appropriate controls and recovery stage comparisons. Still, including initial microbial data would improve the ability to disentangle treatment effects from native dynamics and is recommended for future studies.

## Electronic supplementary material

Below is the link to the electronic supplementary material.

Supplementary Material 1

Supplementary Material 2

## Data Availability

No datasets were generated or analysed during the current study.
