# Supplementary material for: Interaction between arbuscular mycorrhizal fungi and native soil microbiome on early stage restoration of a coal-mine soil
Source: Mycorrhiza. 2025 Aug 8;35(4):49. doi: 10.1007/s00572-025-01218-3 (PMC12334516; doi:10.1007/s00572-025-01218-3)
Supplement: Supplementary file 2 — Supplementary Material 2 [file 572_2025_1218_MOESM2_ESM.docx]

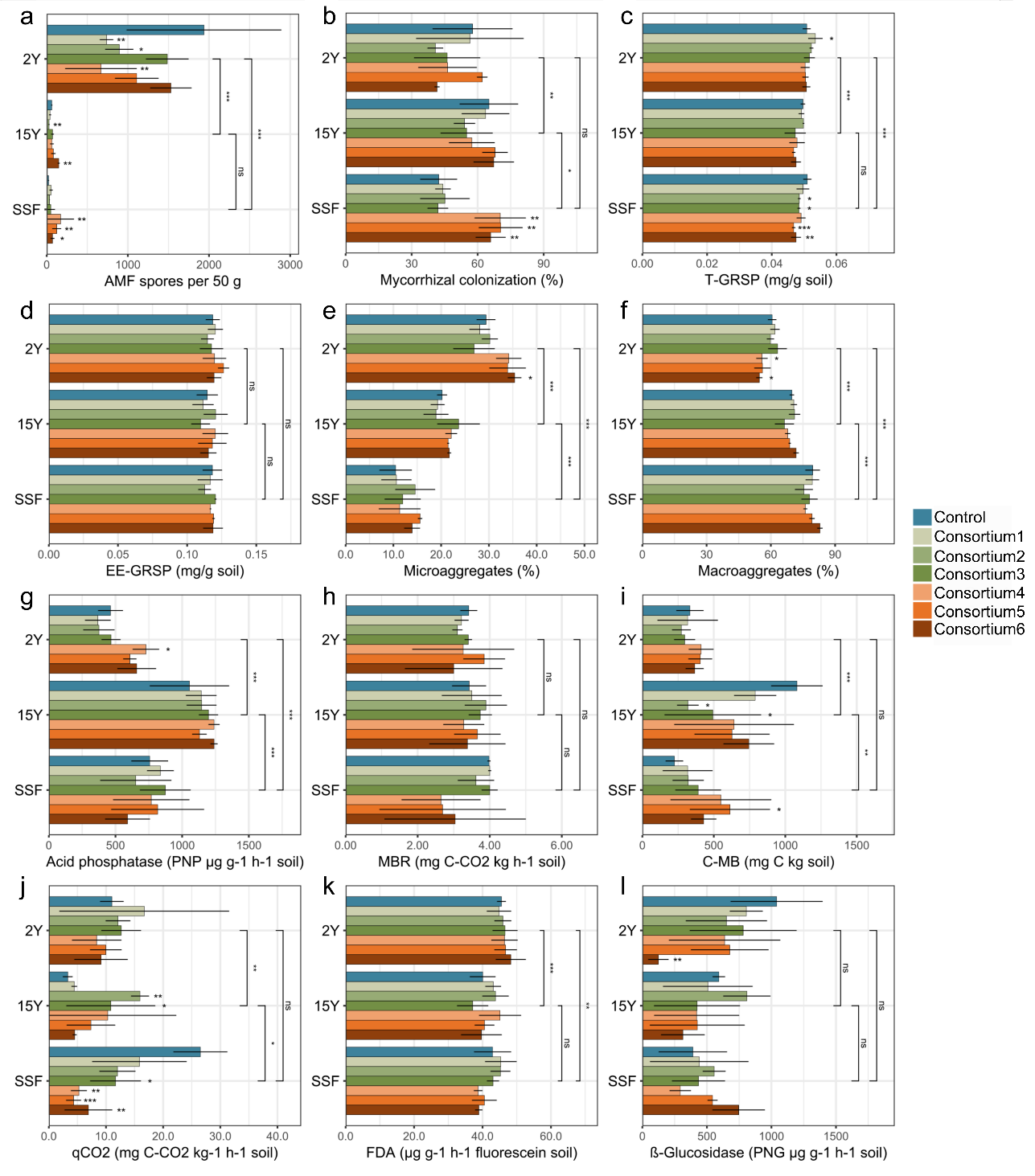


**Fig. S1.** Soil and AM parameters post-greenhouse experiment. Parameters assessed include (A) a percentage of soil macroaggregates, (B) total glomalin-related soil protein (T-GRSP), (C) acid phosphatase activity measured as p-nitrophenyl phosphate (PNP), (D) metabolic quotient (*q*CO_2_), (E) soil microbial biomass carbon (C-MB), (F) macroaggregates, (G) macroaggregates, (H) microbial basal respiration (MBR), (I) soil microbial biomass carbon (C-MB), (J) metabolic quotient (*q*CO_2_), (K) fluorescein diacetate hydrolysis (FDA), and (L) β-glucosidase activity measured as p-nitrophenol. Comparisons were made between control samples and various treatments: AM consortium1 (family Acaulosporaceae), AM consortium2 (family Gigasporaceae), AM consortium3 (families Acaulosporaceae and Gigasporaceae), AM consortium4 (AM fungal richness, n=4), AM consortium5 (AM fungal richness, n=8), and AM consortium6 (AM fungal richness, n=16), within each site: 2 years post-reforestation (2Y), 15 years post-reforestation (15Y), and a secondary succession forest (SSF) as a reference site. Statistical significance between treatments and sites was assessed using ANOVA, with significance levels denoted by asterisks (* *p* < 0.05; ** *p* < 0.01; *** *p* < 0.001). Vertical bars represent contrasts between sites (Two-way ANOVA- Contrast analysis), and standard errors are depicted as black lines.


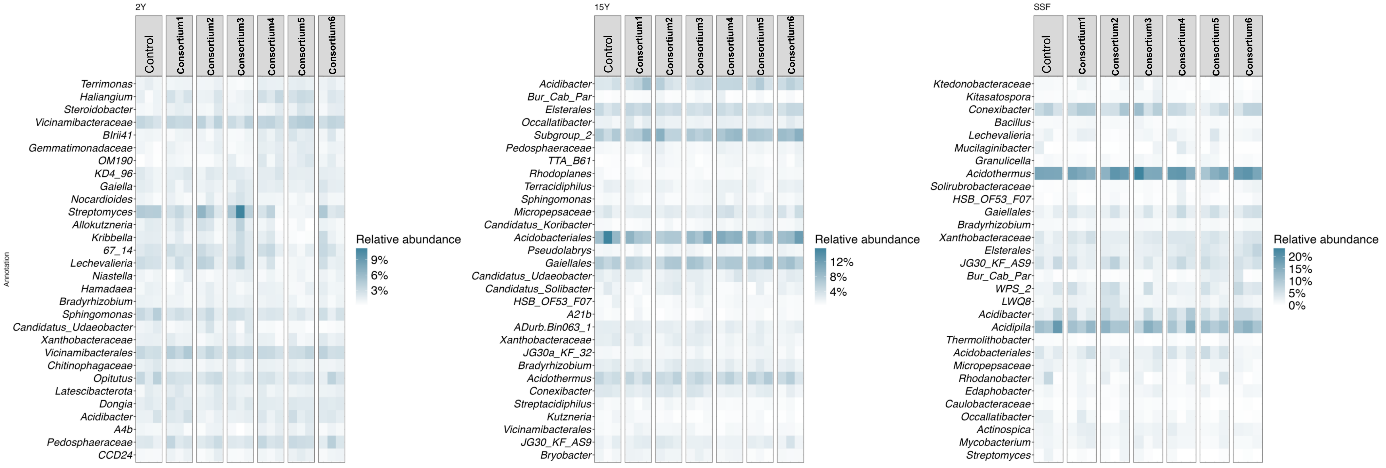


**Fig. S2.** Thirty most abundant taxa based on 16S rRNA gene amplicon sequencing in different treatments, AM consortium1 (Acaulosporaceae), AM consortium2 (Gigasporaceae), AM consortium3 (Acaulosporaceae and Gigasporaceae), AM consortium4 (AM fungal richness, n=4), AM consortium5 (AM fungal richness, n=8), and AM consortium6 (AM fungal richness, n=16), within each site, short-term recovery (2Y), long-term recovery (15Y), and a secondary succession forest (SSF)
